# 3D Reconstruction of the Morpho-Functional Topography of the Human Vagal Trigone

**DOI:** 10.3389/fnana.2021.663399

**Published:** 2021-04-16

**Authors:** Aron Emmi, Andrea Porzionato, Martina Contran, Enrico De Rose, Veronica Macchi, Raffaele De Caro

**Affiliations:** Department of Neuroscience, Institute of Human Anatomy, University of Padua, Padua, Italy

**Keywords:** neuroanatomy, vagal trigone, dorsal motor nucleus of the vagus, solitary tract nucleus, 3D reconstruction, 3D printing

## Abstract

The Vagal Trigone, often referred to as Ala Cinerea, is a triangular-shaped area of the floor of the fourth ventricle that is strictly involved in the network of chardiochronotropic, baroceptive, respiratory, and gastrointestinal control systems of the medulla oblongata. While it is frequently identified as the superficial landmark for the underlying Dorsal Motor Nucleus of the Vagus, this correspondence is not univocal in anatomical literature and is often oversimplified in neuroanatomy textbooks and neurosurgical atlases. As the structure represents an important landmark for neurosurgical procedures involving the floor of the fourth ventricle, accurate morphological characterization is required to avoid unwanted side effects (e.g., bradychardia, hypertension) during neuorphysiological monitoring and cranial nerve nuclei stimulation in intraoperative settings. The aim of this study was to address the anatomo-topographical relationships of the Vagal Trigone with the underlying nuclei. For this purpose, we have conducted an anatomo-microscopical examination of serial sections deriving from 54 Human Brainstems followed by 3D reconstruction and rendering of the specimens. Our findings indicate that the Vagal Trigone corresponds only partially with the Dorsal Motor Nucleus of the Vagus, while most of its axial profile is occupied by the dorsal regions of the Solitary Tract Nucleus. Furthermore, basing on literature and our findings we speculate that the neuroblasts of the Dorsal Motor Nucleus of the Vagus undergo neurobiotaxic migration induced by the neuroblasts of the dorsolaterally located solitary tract nucleus, giving rise to the Ala Cinerea, a topographically defined area for parasympathetic visceral control.

## Introduction

The Vagal Trigone, also known as Ala Cinerea, is a topographical region of the Human Rhomboid Fossa located laterally to the Hypoglossal Trigone and medially to the Vestibular Trigone.

These medullary trigones represent important surface landmarks at the level of the rhomboid fossa during the dorsal approach to intraaxial pontomedullary lesions or during the ultimate exposure of the fourth ventricular floor as an intraventricular tumor is excised (Skinner, 2011). However, as visualized anatomy alone cannot be entirely relied upon before brainstem incision (Skinner, 2011) due to possible distortions given by intraventricular lesions or intraaxial tumors (Morota and Deletis, 2006), intraoperative electrophysiological stimulation, and monitoring is employed to map the caudal rhomboid fossa and to assess nuclear integrity, particularly at the level of the Hypoglossal and Vagal Trigone. During stimulation, particular care must be taken to avoid injury to brainstem structures or excessive stimulation of vagal nuclei, which can lead to severe bradycardia or abrupt blood pressure changes: the vagal trigone is, in fact, topographically and functionally involved in the network of cardiochronotropic, baroceptive, respiratory, and gastrointestinal control systems of the medulla oblongata (De Caro et al., 2000; Travagli and Anselmi, 2016; Porzionato et al., 2019).

Although both neuroanatomy textbooks and neurosurgical atlases (e.g. Linn et al., 2009; Naidich et al., 2009; Skinner, 2011; Vanderah and Gould, 2015; Standring, 2018; Mirza and Das, 2020) identify a direct correspondence between the Vagal Trigone and the Dorsal Motor Nucleus of the Vagus, the topographical description of the district varies noticeably in literature. Furthermore, the anatomical terminology of these structures, with particular regard to the Dorsal Motor Nucleus of the Vagus (DMNV) and the Solitary Tract Nucleus (STN), does not appear to be univocal.

While the detailed delineation of the boundaries and subnuclear divisions of brainstem nuclei has already been characterized in literature, the relationship between superficial landmarks (such as the Vagal Trigone) and underlying structures has been investigated only in few studies, mostly based on observations deriving from a single specimen (Streeter, 1903; Weed, 1914). For this purpose, we have reviewed the scientific literature regarding the anatomical organization of the rhomboid fossa and conducted an anatomo-topographical study of serial sections of the human brainstem deriving from 54 Body Donors of the Body Donation Program of the University of Padua.

### Background and Brief Review of Literature

The relationship between the morphology of the rhomboid fossa and the underlying nuclei of the brainstem tegmentum has been a subject of investigation since the early studies of Friederich Arnold and Benedikt Stilling (Stilling, 1842). With particular regard to the Vagal Trigone, Stilling described three triangular areas on each side of the dorsal median sulcus at the level of the caudal part of the rhomboid fossa: the Hypoglossal Trigone, the Vagal Trigone (Ala Cinerea), and the Vestibular Trigone. The Author first identifies the Vagal Trigone (Ala Cinerea) as a dark colored triangular area, hence the latin term “*cinerea*” (ashen, gray), extending from the Calamus Scriptorius, medially, to the acoustic striae, laterally.

According to Stilling, the Vagal Trigone is divided by a small ridge at the level of the base of the triangle, identifying anteriorly the area corresponding to the nucleus of the Vagus Nerve (X), and posteriorly the area of the nucleus of the Accessory Nerve (XI).

In 1896, Magnus Gustaf Retzius provides the most detailed description of the human rhomboid fossa, which is still largely used in modern anatomy textbooks (Retzius, 1896; Streeter, 1903). According to the Author, the Ala Cinerea is located laterally to the hypoglossal trigone and medially to the area acustica (comprising the vestibular trigone). The Vagal Trigone is separated from the caudally located Area Postrema by a well-marked ridge of ependyma, known as “Funiculus Separans,” and extends forward between the area acustica and the trigonum hypoglossi, taking on the shape of a triangle with inferior base. According to Retzius (1896), Streeter (1903), and Weed (1914), the Ala Cinerea corresponds to the underlying Nucleus of the Ala Cinerea.

According to Dejerine (1902) there is a topographical correspondence between the Fasciculus Separans and the Solitary Tract. This, however, has not been found in Streeter's study (1903), who evidenced no apparent relation between the two structures.

In the work of Chiarugi and Bucciante (1965), the Authors identify two main nuclei at the level of the Ala Cinerea: the dorsal parasympathetic motor nucleus (dorsal motor nucleus of the vagus), visceromotor, and the nucleus of the Ala Cinerea, viscerosensory. The latter is described as lateral to the dorsal parasympathetic motor nucleus, and appears to be distinct from the solitary tract and the STN, which is described more ventrally; the STN described by the Authors corresponds exclusively to the mantle of gray matter strictly surrounding the solitary tract, with the Nucleus of the Ala Cinerea being considered as a separate structure. More recently McRitchie and Törk (1993) performed a detailed delineation of the outer boundaries and subnuclear divisions of the human STN, correlating the subdivisions to their homologs described in animals. Furthermore, the boundaries of the DMNV and of the other nuclei of the brainstem tegmentum were clearly delineated in humans by Paxinos and Huang (1995).

Within this context, it is necessary to define the relationship between the surface morphology of the rhomboid fossa and the underlying nuclei of the brainstem tegmentum in light of the more recent definition of the boundaries of these structures, not only for their proper description in neuroanatomy textbooks (Standring, 2018), but also for their relevance in neurosurgery and neurophysiological monitoring (e.g. Skinner, 2011).

## Materials and Methods

**Figure 1:**
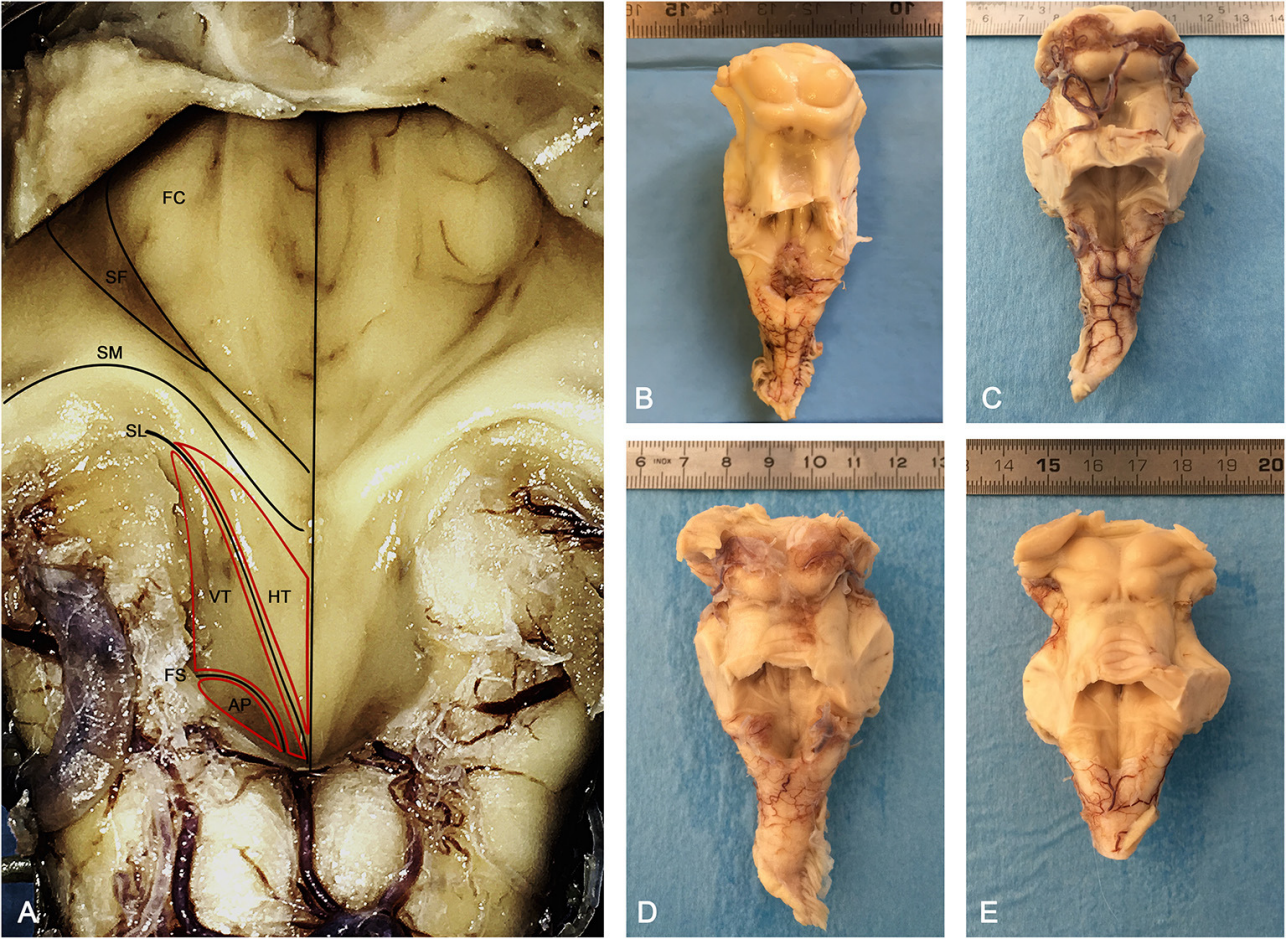
Morphology of the human rhomboid fossa. **(A)** Structures and landmarks of the rhomboid fossa. The Vagal Trigone (Ala Cinerea) appears as a triangular structure emerging from the Calamus Scriptorius and ending with its apex at the level of the Striae Medullares; caudally it is separated from the Area Postrema by the Funiculus Separans. AP, Area Postrema; FS, Funiculus Separans; VT, Vagal Trigone (Ala Cinerea); HT, Hypoglossal Trigone (Ala Bianca Interna); SL, Sulcus Limitans; SM, Striae Medullares (Acustic Striae of Piccolomini); SF, Superior Fovea; FC, Facial Colliculus. **(B–E)** Posterior view of four brainstems employed in the study, anatomically sectioned at the level of the mesencephalon, cranially to the superior colliculi, and at the level of the cerebellar pedicles.

**Figure 2:**
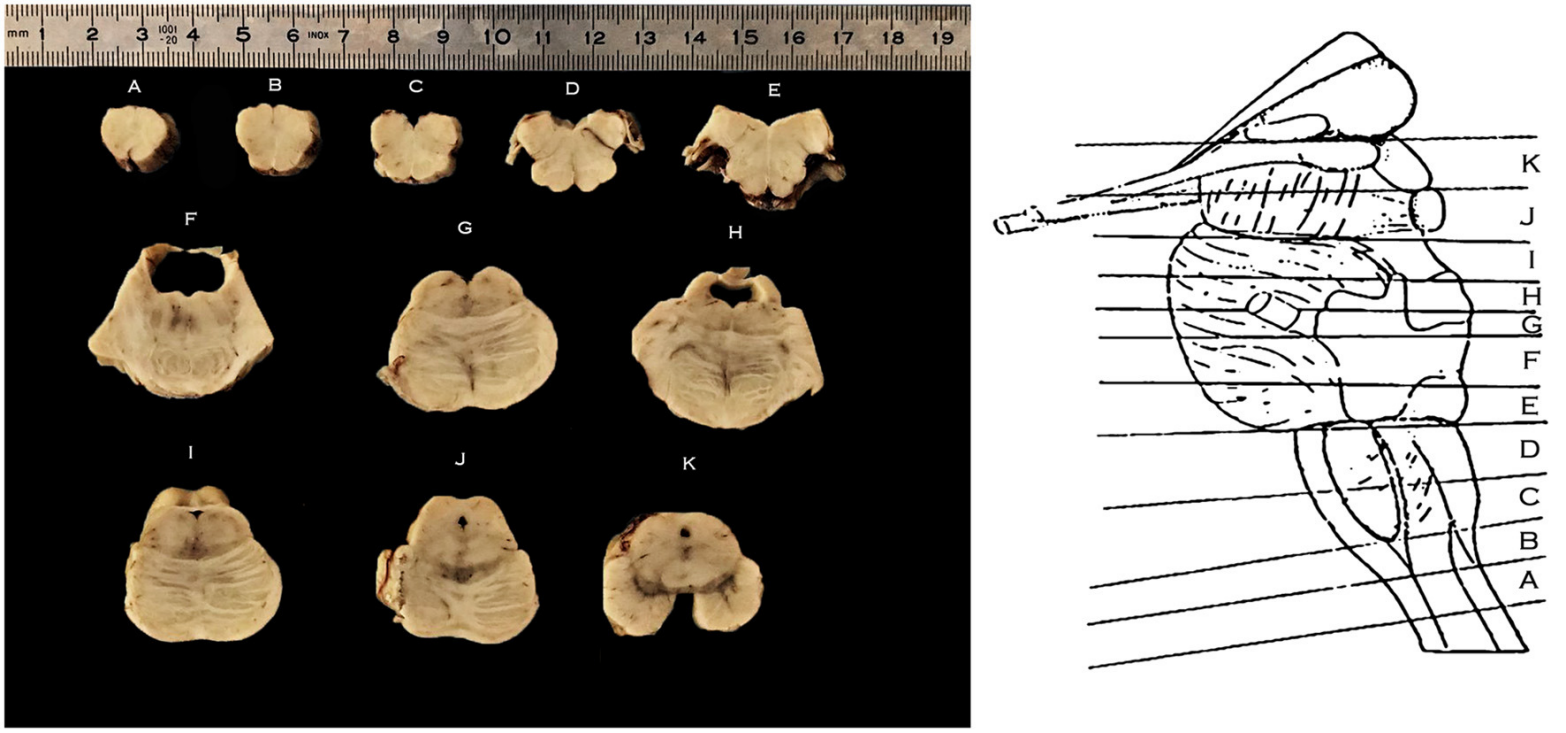
Right, schematic representation of the levels of sectioning of the Human Brainstem, lateral perspective following isolation of the cerebellum; Left, axial sections of the brainstem according to the schema on the right; caudal surface facing upwards. From caudal to cranial: **(A)** section at the level of the decussation of the pyramids; **(B)** section at the level of the sensory decussation; **(C)** section at the level of the inferior half of the medullary olivary complex; **(D)** section at the level of the superior half of the medullary olivary complex; **(E)** section at the level of the caudal pons and the colliculus facialis; **(F,G)** section at the level of the intermediate pons; **(H,I)** section at the level of the cranial pons; **(J)** section at the level of the mesencephalo-pontine sulcus; **(K)** section at the level of the mesencephalon.

Fifty-four Human Brains with no history of neurological or psychiatric disorders deriving from the Body Donation Program of the University of Padua (Italy) (Porzionato et al., 2012; Boscolo-Berto et al., 2020) (mean age 63.4 years) were employed for the topographic examination of the rhomboid fossa. The brains were fixed in-toto in 4% phosphate-buffered paraformaldehyde for at least 3 weeks and subsequently sectioned in order to anatomically isolate the brainstem from the cerebrum at the level of the mesencephalon. The brainstem was then isolated from the cerebellum by sectioning the cerebellar pedicles in proximity to the cerebellar hylum. The choroid plexus of the fourth ventricle was then removed in order to clearly evaluate the morphology of the rhomboid fossa (Figure 1). Axial sections of the brainstem were then conducted perpendicular to the floor of the fourth ventricle, according to the schema in Figure 2.

Scaled photographs were taken of the cut surface prior and following tissue processing, in order to evaluate the amount of shrinkage induced by further processing according to previously published protocol (Porzionato et al., 2020a). Tissue sections were dehydrated, cleared in xylene, and embedded in paraffin. Since the Ala Cinerea was contained in most specimen in sections B–D, these were serially sectioned in 10-μm-thick axial sections using a calibrated rotatory microtome (RM 2235, Leica Microsystems, Wetzlar, Germany). Disposable metal microtome blades (Bio-Optica, Milan, Italy) were used with a cutting angle of about 5°. Systematic and uniform random sampling of the sections containing the Ala Cinerea was performed. In each specimen, three consecutive series of adjacent sections were taken at 1/10 interval, and stained (1) for Haematoxylin-Eosin (2) Cresyl Violet (Nissl), and (3) Luxol-Van Gieson stain.

Color images of the consecutive sections were acquired using a digital camera (Optikam HDMI, Optika srl, Italy), attached to a stereo microscope (SZM-2, Optika srl, Italy) operating with transmitted light at a primary magnification of x0.67. This allowed to acquire the whole profile of the sections for 3D reconstruction purposes.

Microscopic examination of the sections was performed under a Leica DM4500B microscope (Leica Microsystems) equipped with a motorized object table and a microcator with digital readout for measuring movements in the Z-direction with 0.5 μm precision. The microscope was connected to a Leica DFC320 high-resolution digital camera (Leica Microsystems) and a computer equipped with softwares for image acquisition (QWin, Leica Microsystems) and analysis (ImageJ).

**Figure 3:**
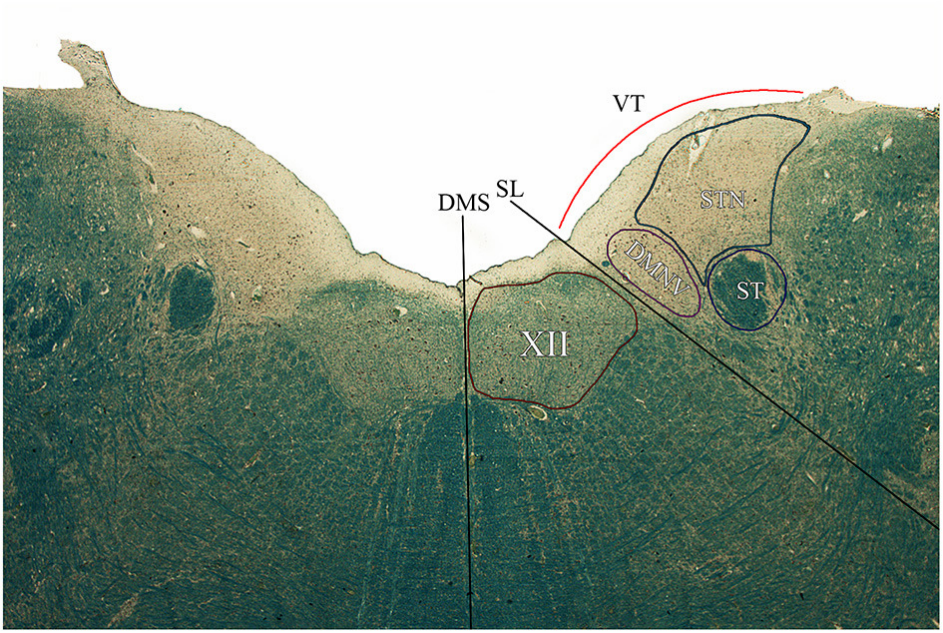
Axial section of the brainstem tegmentum at the level of section C according to the Schema in Figure 2. DMS, dorsal median sulcus; SL, Sulcus Limitans; VT, Vagal Trigone; XII, Hypoglossal Nucleus; DMNV, Dorsal Motor Nucleus of the Vagus; ST, Solitary Tract; STN, Solitary Tract Nucleus.

The profile of the reliefs of the rhomboid fossa, along with the boundaries of the hypoglossal nucleus, dorsal motor nucleus of the vagus, solitary tract, and solitary tract nucleus were defined on 1.25x magnification according to Mai and Paxinos (2012) and drawn by three independent anatomists (RDC, AP, AE) employing ImageJ software, as seen in Figure 3. Briefly, Atlas boundaries and histochemical images were confronted with on-screen digital images of the specimen. Criteria for discerning neurons belonging to the different nuclei were identified as (1) topographical location along the medio-lateral axis; (2) average cell size; and (3) peculiar characteristics of specific nuclei defined in past anatomical literature (i.e., hyaline cytoplasmatic inclusions in hypoglossal neurons, pigmented neurons intercalated in STN, etc). Emphasis was set on the relationship between surface landmarks and underlying nuclear masses.

3D reconstruction procedures were employed on every specimen at the level of sections B–D, while one brasinstem was completely sectioned in its whole cranio-caudal extent and reconstructed in-toto for representation purposes.

**Figure 4:**
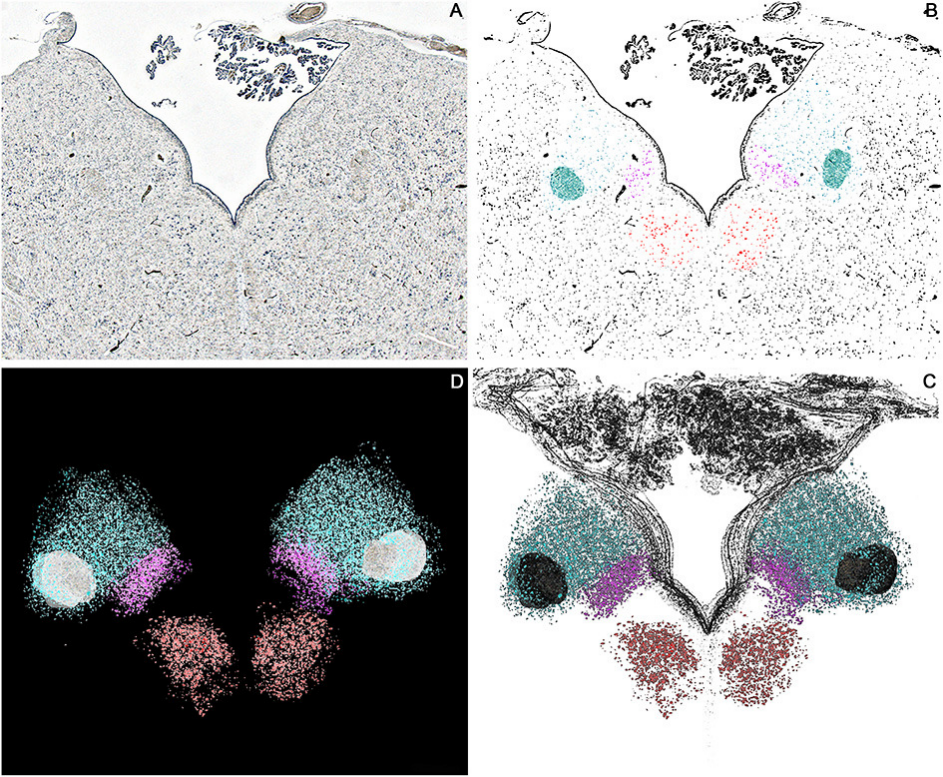
Axial sections of one brainstem specimen at the level of the Ala Cinerea (Tissue sections **(B,C)** according to Figure 1). **(A)** Nissl Stained section, 1.25x Magnification. **(B)** Thresholded photomicrograph with color-labeled Regions of Interest (ROIs) for the Hypoglossal Nucleus (Red), Dorsal Motor Nucleus of the Vagus (Violet), Solitary Tract and Solitary Tract Nucleus (Blue). **(C)** 3D reconstruction of the neuronal profiles of the Hypoglossal Nucleus (Red), Dorsal Motor Nucleus of the Vagus (Violet), Solitary Tract, and Solitary Tract Nucleus (Blue) at the level of the Ala Cinerea [Tissue sections **(B,C)**]. **(D)** 3D Reconstruction of the Rhomboid Fossa along with the neuronal populations of the nuclei of the brainstem tegmentum (Hypoglossal Nucleus in Red; Dorsal Motor Nucleus of the Vagus in Violet; Solitary tract and nucleus in Blue).

**Figure 5:**
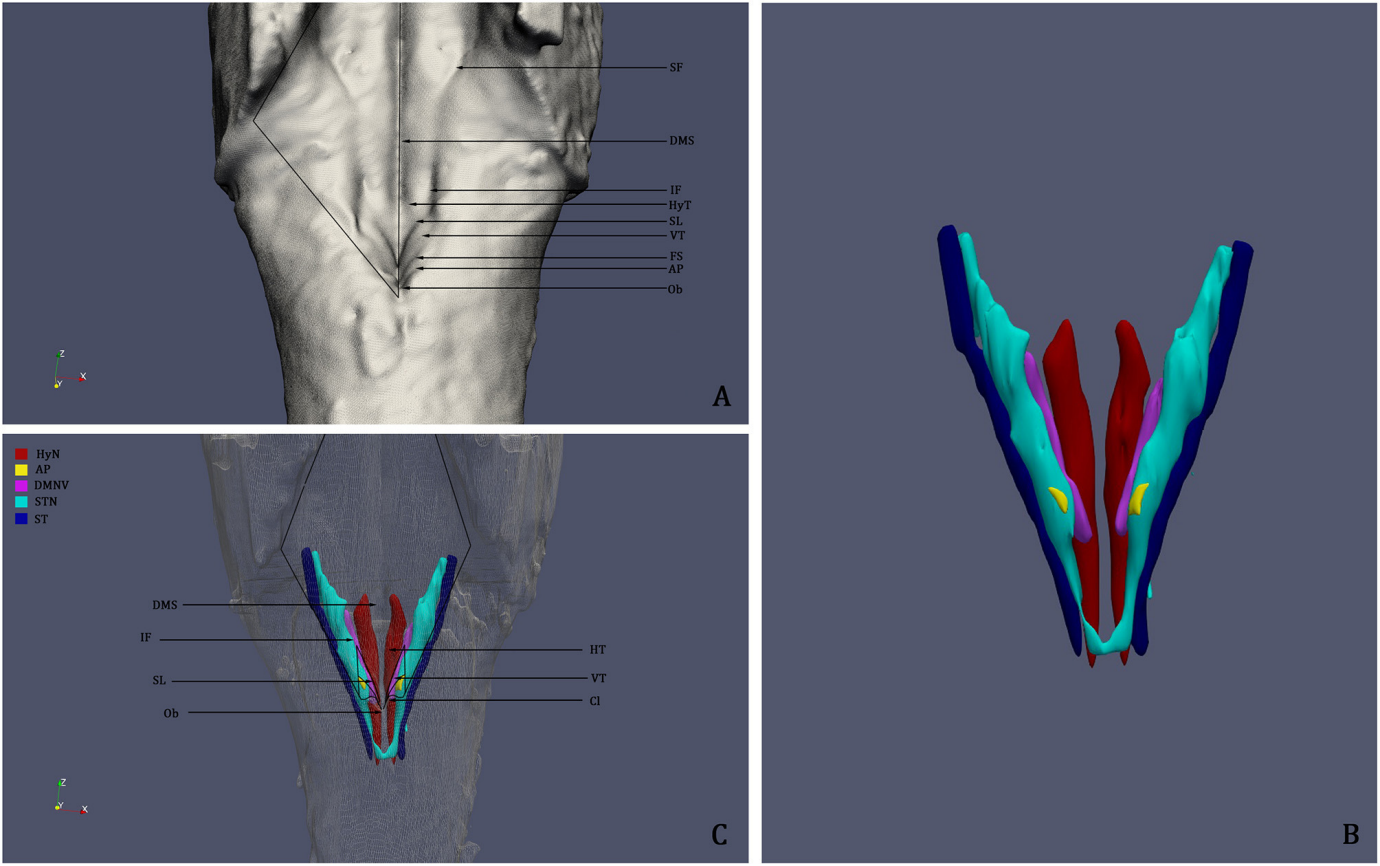
**(A)** 3D reconstruction and rendering of the Human Brainstem, perspective on the Rhomboid Fossa. **(B)** 3D reconstruction of the nuclei of the brainstem tegmentum; **(C)** Surface projection of the nuclei of the tegmentum on the floor of the fourth ventricle; the rendering clearly shows that the Vagal Trigone (VT) contains both the Dorsal Motor Nucleus of the Vagus and the Solitary Tract Nucleus. DMS, Dorsal Median Sulcus; SF, Superior Fovea; IF, Inferior Fovea; SL; Sulcus Limitans; FS, Fasciculus Separans; HT, Hypoglossal Trigone; VT, Vagal Trigone; Ob, Obex; HyN, Hypoglossal Nucleus; AP, Area Postrema; DMNV, Dorsal Motor Nucleus of the Vagus; STN, Solitary Tract Nucleus; ST, Solitary Tract.

Images of the consecutive sections were registered and aligned employing the Marquardt-Levenberg algorithm for non-linear least-squares optimization (Porzionato et al., 2020b). Cresyl violet stained sections were segmented through manual thresholding in order to isolate the neuronal somata (violet) from the background (unstained); thresholded images were then confronted with the original photomicrographs to ensure that no relevant elements were lost and to define the previously drawn boundaries. Hypoglossal nucleus, DMNV, Area Postrema, STN, and ST were defined as Regions of Interest (ROI) and coded with different colors (Figures 4, 5).

Three-dimensional surface rendering was performed using the Visualization Toolkit (VTK, version 7.0), an open source library developed by Kitware Inc. (New York, USA) freely available at http://www.vtk.org. The 3D surfaces of the brainstem and of the defined ROIs were smoothed with a Gaussian Filter with 0.3 intensity and saved as.vtk files, which were then rendered as a single scene in the Paraview software (version 5.0.1, Kitware Inc.). Labeling of surface structures was performed according to Paxinos and Huang (1995) and the review of literature.

**Figure 6:**
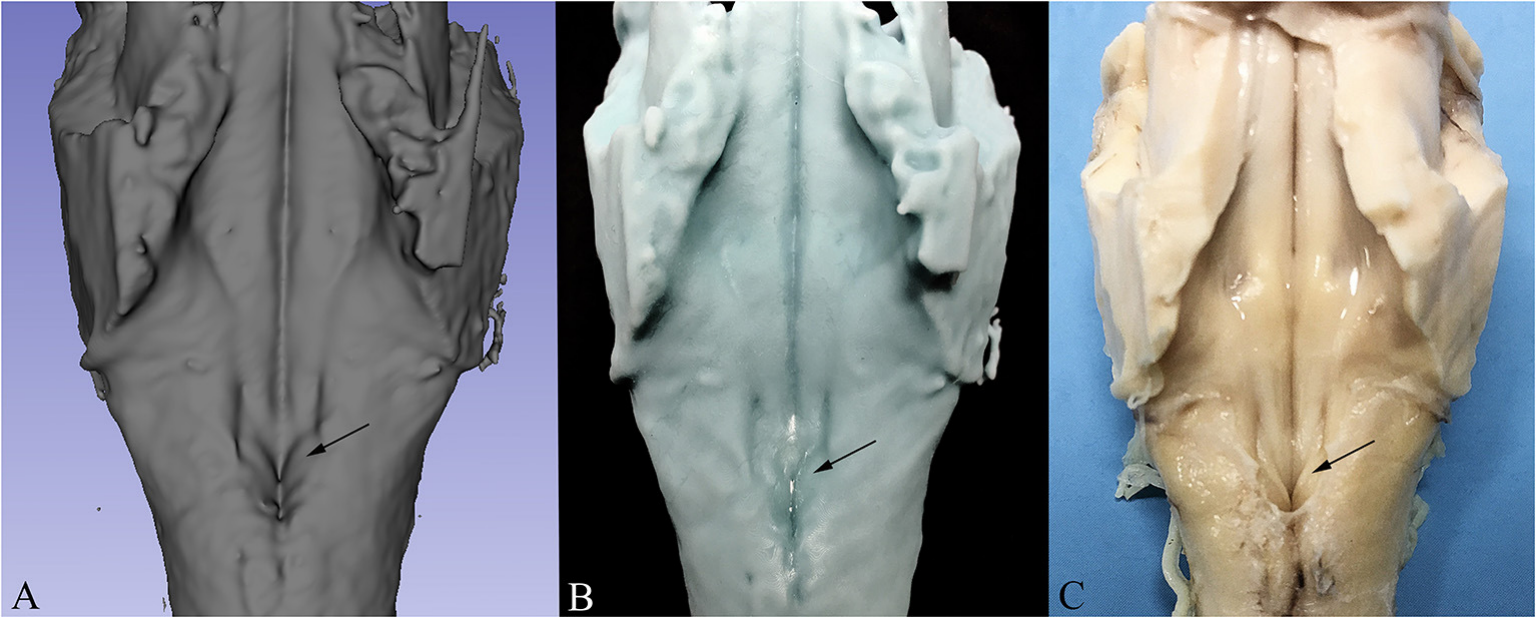
Comparison between 3D Reconstruction **(A)**, 3D Print based on the reconstructed model **(B)** and the real specimen prior to sectioning **(C)**. The arrow indicates the Vagal Trigone in all three images.

In order to compare the virtual reconstruction to the real specimen prior to sectioning, 3D printing was performed on the in-toto reconstructed specimen in order to re-create the physical structure of the examined brainstem and to evaluate the quality of the reconstruction. For this purpose, Digital Light Processing 3D Printer (Elegoo Mars 3D Printer) was employed. Digital Light Processing based machines are used to create small models that require high resolution (10 μ on Z axis and 47 μ on X/Y axis) with a UV-sensitive (405 nm) liquid resin. The resulting model can be appreciated in Figure 6.

## Results

Upon macroscopic examination of the floor of the fourth ventricle, 47 (87.1%) specimen presented a slightly dark colored Ala Cinerea, with a typical triangular profile with the base emerging from the calamus scriptorius and the apex pointing toward the acoustic striae (Figure 1). In the remaining specimen (12.9%) the Ala Cinerea was also morphologically identifiable but presented a less prominent dark coloration.

The Vagal Trigone was found laterally to the Hypoglossal Trigone and medially to the Vestibular Trigone, as described by previous authors.

By comparing the boundaries identified by McRitchie and Törk (1993) and Paxinos and Huang (1995) with the nuclei described by the authors of the late nineteenth and early twentieth century (e.g., Stilling; Retzius; Streeter; Weed; Dejerine), it appears that: (a) the nucleus of the ala cinerea described by Stilling (1842), Streeter (1903), and Weed (1914) corresponds to Paxinos and Huang (1995) DMNV; (b) the nucleus of the ala cinerea described by Chiarugi and Bucciante (1965) corresponds to the medial subnucleus of the STN, while the STN defined by Chiarugi and Bucciante (1965) corresponds to the interstital, ventral, and ventrolateral subnuclei of the STN described by Paxinos and Huang (1995).

Microscopic examination of brainstem serial sections (sectioned according to the schema in Figure 2) revealed a topographical correspondence between the axial profile of the Vagal Trigone and the parasympathetic viscerosensory nuclei of the medullary tegmentum. In particular, both the DMNV and the STN were found within the axial surface of the Vagal Trigone. The DMNV was located medially, at the boundary between the Vagal Trigone and the Hypoglossal Trigone, while the STN was found laterally to the DMNV, representing most of the underlying gray matter of the Vagal Trigone (approximately two-thirds of the structure). The STN extended, in every specimen, from the DMNV to the medial border of the vestibular complex, as seen in Figure 3, and is represented caudally by the dorsolateral subnucleus, and rostrally by the dorsal subnucleus. Neurons of both DMNV and STN nuclei were found in close proximity to the ependymal surface of the fourth ventricle.

The sulcus limitans was clearly defined and identifiable in 47 specimen (87.1%), with a minor percentage showing a more flat profile of the floor of the fourth ventricle in microscopic examination; these findings match the macroscopic examination prior to sectioning, as stated above. In every specimen with a clearly marked sulcus limitans, the DMNV was located laterally, and in close proximity, to the sulcus itself. In a smaller percentage of specimen (eight specimen, i.e., 14.8% of the total sample) a superficial depression could be identified laterally to the DMNV. This depression, which did not match to the funiculus separans or any other previously described sulci, appeared to partially mark the superficial territory of the DMNV from the STN in these specimen. Laterally, the Ala Cinerea was bounded by a vertically descending sulcus, separating it from the vestibular trigone. This less prominent sulcus was evident 24 specimen (44%), while in the remaining percentage appeared as a less prominent depression at the level of the boundary between the lateral fringe of the Solitary Tract Nucleus and the nuclei of the vestibular trigone. When present, the sulcus originated cranially as a branching of the sulcus limitans, marking the boundary between Ala Cinerea and Vestibular Trigone.

3D reconstruction confirmed the aforementioned microscopic findings (Figures 4–6). In Figure 4, the axial perspective of the reconstruction identifies a correspondence between the Ala Cinerea and the DMNV (occupying the medial third of the Ala cinerea) and the STN (occupying the lateral two-thirds of the structure). Figure 5A shows the 3D rendering of the reconstructed brainstem, rendered in order to highlight surface morphology, while Figure 5B shows the segmented ROIs corresponding to the nuclei of the brainstem tegmentum. In Figure 5C, the surface projection of the aforementioned nuclei clearly shows the correspondence between the Ala Cinerea and the DMNV and STN, with the sulcus limitans and the funiculus separans separating the vestibular trigone from the hypoglossal trigone and the area postrema, respectively. 3D reconstruction of specimen presenting the accessory depression laterally to the DMNV did not reveal an appreciable landmark in surface morphology, being only visible in transverse sections during microscopic examination. 3D printing revealed good correspondence between the actual specimen and the reconstructed and 3D printed model, according to Figure 6.

## Discussion

The relationship between surface morphology and underlying brainstem nuclei arises from an historical interest that finds modern applications in neurosurgical procedures and neuroanatomical education.

The Vagal Trigone is an important landmark of the human rhomboid fossa, as it contains the main parasympathetic visceromotor and viscerosensory nuclei of the brainstem. The trigone is morpho-functionally embedded into the network of cardiochronotropic, baroceptive, and respiratory control systems of the medullary tegmentum, as well as gastrointestinal regulatory circuits (Browning and Travagli, 2019). With particular regard to cardiovascular control systems, the STN receives baroreceptor afferences from the carotid sinus nerve (Porzionato et al., 2019), while the nucleus ambiguus outputs cardioregulatory efferents, with a minor contribution from the DMNV. Electrical stimulation or mechanical injury during neurosurgical procedures at the level of the Vagal Trigone may lead to bradycardia, hypotension, or severe hypertension, particularly when the STN is involved (Colombari et al., 2001; Dampney et al., 2003; Skinner, 2011). Gastrointestinal dysfunction determined by vagal trigone injury must also be considered, as it may represent a relevant post-operatory concern given the crucial role of the DMNV and STN in viscerosensory and visceromotor control of the alimentary tract (Mann et al., 1998; Travagli and Anselmi, 2016). Hence, accurate knowledge of the topography of the region is crucial in avoiding severe unwanted side effects during neurosurgery.

Embryologically, the sulcus limitans represents the primordial boundary between the motor Basal Plate and the sensory Alar Plate (Mai and Paxinos, 2012). In the adult spinal cord, the orthosympathetic visceromotor and general viscerosensory centers are found at the level of the boundary between the basis of the ventral and dorsal horn (Rexed Lamina 7 and 10) (Mai and Paxinos, 2012), with the viscerosensory centers being located more medially (intermediomedial nucleus) and the visceromotor centers (intermediolateral nucleus) being located laterally and matching the profile of the lateral horn. Similarly, at the level of the rhomboencephalon, the visceromotor and viscerosensory centers are located at the boundary between the derivates of the Basal Plate and the Alar Plate, in close proximity to the sulcus limitans, with the visceromotor DMNV being more ventro-medial and the viscerosensory STN being more dorso-lateral.

The peculiar disposition of these structures at the level of the rhomboencephalon, compared to their spinal cord homologs, can be inferred by the embryonal development of the rhombonecephalon. Starting from the 6th week of development, the basal plate differentiates into three distinct columns, with the most lateral one becoming the general visceral efferent column and giving rise to the DMNV at the level of rhombomers 7–8. In particular, ventrally migrating neuroblasts of the basal lamina give rise to the nucleus ambiguus (or Somatic Branchial Column), dorsally migrating neuroblasts give rise to the DMNV (General Visceromotor Column), while the Somatic Somitic Column (Hypoglossal Nucleus) remains in proximity of the midline. Conversely, the Alar Plate gives rise to the general visceral afferent column, being separated from the ventro-medially located general visceral efferent column by the sulcus limitans.

This disposition, which reflects the organization of the rhomboid fossa during early development, and in particular before the 9th gestational week (Paxinos and Huang, 1995; Cheng et al., 2004; Mai and Paxinos, 2012), does not appear to entirely match the topographical disposition of the visceromotor and viscerosensory centers of the brainstem tegmentum in adults, as testified by our current findings: the DMNV appears to be located laterally to the sulcus limitans, forming the Vagal Trigone along with the STN.

Interestingly, Cheng et al. (2004) used carbocyanine tracing to label DMNV neurons in the developing fetus (9th to 28th gestational week), identifying numerous labeled cells in proximity to the sulcus limitans. By closely examining the figures and the data reported in Cheng et al.'s work, it appears that: (a) the DMNV is located medially to the sulcus limitans in the 9 g.w. specimen and that (b) the same nucleus is found laterally to the sulcus limitans in older specimen (from g.w. 13th to 28th).

The dorsolateral migration of DMNV neuroblasts is also supported by morphological and pathological findings in cases of Sudden Infant Death Syndrome (Macchi et al., 2002). In these conditions, incomplete migration of the DMNV neuroblasts gives rise to morphological alterations in the brainstem tegmentum, such as hypocellular, more-medially located DMNV and hypercellular homolateral hypoglossal nucleus, possibly leading to abnormal autonomic cardio-respiratory control.

Together with our findings it could be speculated that neurons of the DMNV migrate dorsolaterally toward the STN starting from the 9th g.w. onwards, positioning laterally to the sulcus limitans. This phenomenon, which may be ascribed to neurobiotaxis, a chemotactic effect of visceral sensory neuroblasts on visceromotor neuroblasts, can explain the topography of the DMNV in adult humans, as evidenced by our anatomo-topographical investigation.

These events eventually lead to the morphological delineation of the Ala Cinerea, a specialized and topographically defined area for parasympathetic visceral control that, unlike its oversimplified representation in neuroanatomy textbooks and atlases, does not correspond entirely with the DMNV, but actually comprises the dorsal parts of the STN for two-thirds of its axial profile.

## Conclusions

In the present paper, we present the anatomo-topographical organization of the nuclei underlying the human vagal trigone, or Ala Cinerea, following microscopical examination and 3D reconstruction of 54 brainstems. Our findings indicate that in every examined specimen, the Dorsal Motor Nucleus of the Vagus is located laterally and in close proximity to the sulcus limitans and forms the Vagal Trigone together with the STN, which occupies the lateral two-thirds of the axial profile of this structure.

The relevance of this study is not merely morphological, but resides in the anatomo-functional and clinical significance of the parasympathetic control centers in the brainstem, which are often oversimplified in neuroanatomy textbooks and represent an important landmark for neurophysiological monitoring during surgical procedures. While electrophysiological stimulation and recording are crucial for mapping the caudal rhomboid fossa during brainstem neurosurgery, particular care must be taken when evaluating the vagal trigone. Unlike its typical depiction in literature, only the medial third of the trigone corresponds to the DMNV, while the remaining lateral two-thirds corresponds to the dorsal and dorsolateral regions of the STN. According to our findings, the most constant superficial landmark for the identification of the underlying DMNV is represented by the sulcus limitans, which is located medially and in close proximity to the nucleus. As excessive electrical stimulation or injury to the STN is known to cause severe, at times irreversible, hypertension, bradychardia, and gastrointestinal dysfunction (Colombari et al., 2001; Dampney et al., 2003; Skinner, 2011), anatomical knowledge of the district appears crucial to avoid unwanted intra and postoperatory side effects given by injury to the areas of vagal trigone occupied by the STN.

Furthermore, previous studies have examined the role of the STN in acute heart failure (De Caro et al., 2000), which is anatomically located in a watershed zone that represents the last meadow (i.e., the periphery of a vascular territory vulnerable to low blood perfusion) in the case of sudden fall of the systemic blood flow due to acute heart failure. The STN and the DMNV have also received significant interest in COVID-19 research (Porzionato et al., 2020c), with particular regard to viral neurotropism. Considering the anatomical, clinical, and surgical relevance of these structures, and their peculiar organization in the human brainstem, detailed anatomo-topographical definition may represent a reference basis for further applied and clinical research, as demonstrated for other neuroanatomical structures (Emmi et al., 2020).

## Data Availability Statement

The raw data supporting the conclusions of this article will be made available by the authors, without undue reservation.

## Ethics Statement

Ethical review and approval was not required for the study on human participants in accordance with the local legislation and institutional requirements. The patients/participants provided their written informed consent to participate in this study.

## Author Contributions

AE, RD, and AP conceived the study and examined the specimen. AE drafted the manuscript and the figures, and performed the 3D reconstructions. MC, ED, and VM critically reviewed the manuscript. All authors contributed to the final version of the manuscript.

## Conflict of Interest

The authors declare that the research was conducted in the absence of any commercial or financial relationships that could be construed as a potential conflict of interest.
